# Reaction of Chalcones with Cellular Thiols. The Effect of the 4-Substitution of Chalcones and Protonation State of the Thiols on the Addition Process. Diastereoselective Thiol Addition

**DOI:** 10.3390/molecules26144332

**Published:** 2021-07-17

**Authors:** Fatemeh Kenari, Szilárd Molnár, Pál Perjési

**Affiliations:** 1 Institute of Pharmaceutical Chemistry, University of Pécs, H-7624 Pécs, Hungary; kenari.fatemeh@pte.hu (F.K.); molnar.szilard@pte.hu (S.M.); 2 Research Institute for Viticulture and Oenology, University of Pécs, H-7624 Pécs, Hungary

**Keywords:** chalcone, glutathione, cysteine, thiols, Michael addition, diastereoselective addition

## Abstract

Several biological effects of chalcones have been reported to be associated with their thiol reactivity. In vivo, the reactions can result in the formation of small-molecule or protein thiol adducts. Both types of reactions can play a role in the biological effects of this class of compounds. Progress of the reaction of 4-methyl- and 4-methoxychalcone with glutathione and *N*-acetylcysteine was studied by the HPLC-UV-VIS method. The reactions were conducted under three different pH conditions. HPLC-MS measurements confirmed the structure of the formed adducts. The chalcones reacted with both thiols under all incubation conditions. The initial rate and composition of the equilibrium mixtures depended on the ratio of the deprotonated form of the thiols. In the reaction of 4-methoxychalcone with N-acetylcysteine under strongly basic conditions, transformation of the kinetic adduct into the thermodynamically more stable one was observed. Addition of S-protonated *N*-acetylcysteine onto the polar double bonds of the chalcones showed different degrees of diastereoselectivity. Both chalcones showed a Michael-type addition reaction with the ionized and non-ionized forms of the investigated thiols. The initial reactivity of the chalcones and the equilibrium composition of the incubates showed a positive correlation with the degree of ionization of the thiols. Conversions showed systematic differences under each set of conditions. The observed differences can hint at the difference in reported biological actions of 4-methyl- and 4-methoxy-substituted chalcones.

## 1. Introduction

**Figure 1:**
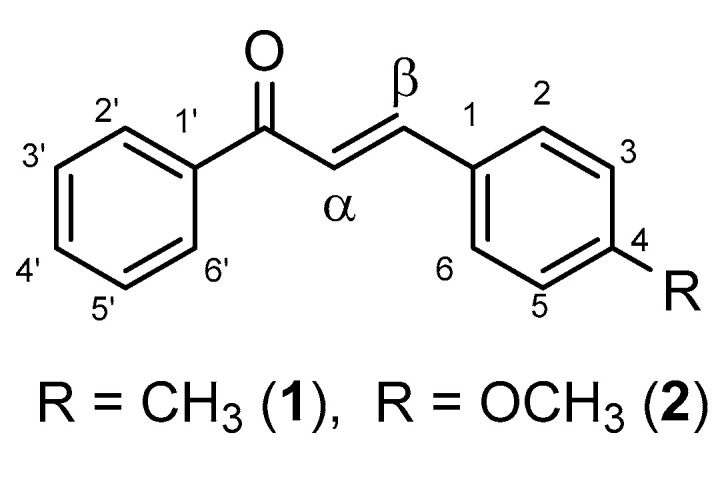
Structure and numbering of the investigated chalcones (**1** and **2**).

Chalcones (Figure 1) are intermediary compounds of the biosynthetic pathway of a large and widespread group of plant constituents known collectively as flavonoids [1]. Several compounds display in vitro cytotoxic (cell growth inhibitor) activity toward cultured tumor cells among the naturally occurring chalcones and their synthetic analogues. Chalcones are also effective as cell proliferation inhibitors and as antitumor-promoting, anti-inflammatory, and chemopreventive agents [2,3,4,5]. Their activity is the result of either covalent or noncovalent interactions [2]. Covalent interactions are mainly based on the Michael acceptor activity of the α,β-unsaturated carbonyl system or the radical-scavenging or reductive potential of the compounds [6,7,8]. Several biological effects (e.g., NQO1 inducer [9], anti-inflammatory [10], GST P1-1 inhibitory [11]) of chalcones have been associated with their Michael-type reactivity toward protein thiols or reduced glutathione (GSH). It was suggested that the lower GSH depletion potential of chalcones with strong electron donor substituents (e.g., dimethylamino) on the B ring could be the consequence of the lower Michael-type reactivity of the derivatives toward GSH [12]. In contrast, higher reactivity toward GSH and other thiols was parallel with the higher NQO1-inducing potential of the investigated chalcones [13].

In our present study, reactions of two chalcones with different 4-substitutions (4-CH_3_ (**1**) and 4-OCH_3_ (**2**)) with two cellular thiols (GSH and NAC) were investigated to get information about (a) how the pH of the reaction medium affects the reactivity and stereochemical outcome of the reactions and (b) how the thiol reactivity of the compounds is related to their biological activities. Since thiol reactivity can be the molecular basis of modulation of the function of thiol switches [14] and the biological effects of covalent modifiers [15], the thiol reactivity of different chalcones is of interest in understanding their biological activities. Glutathione is an endogenous thiol, whose thiol reactivity plays an important role in maintaining redox homeostasis and protecting cellular nucleophilic sites of proteins from harmful electrophiles [16]. NAC is an exogenous thiol; however, it is one of the precursors of GSH biosynthesis, possesses mucolytic action, and is used as an antidote in paracetamol intoxication [17]. Considering their different p*K*_a_ values [18], the two compounds are also perfect models for reactivity screening of compounds with surface protein thiols with different molecular surroundings [19].

The selected substituents have different electron-donating capacities, which results in different reactivities and biological activities. For example, the tumor promotion inhibitory effect of 4-methyl-4′-hydroxychalcone is about twice as high as that of the 4-methoxy analogue [20]. Comparison of the in vitro cytotoxicity of **1** and **2** toward five different cancer cells showed that the 4-methyl derivative (**1**) is more effective toward most of the tested cell lines [21]. Furthermore, multiple regression analysis showed that cytotoxicity of a series of chalcones toward murine and human cancer cells increases as the Hammett sigma (σ) values of the substituent elevates [22]. In contrast, among the cyclic analogues (*n* = 5, 6, and 7) of the two chalcones, the methoxy-substituted (**2**) cyclic derivatives showed much higher in vitro cancer cell cytotoxicity than those of **1** [23]. Furthermore, the seven-membered analogues of **1** and **2** showed different in vitro effects on the cell cycle of Jurkat T cells [24].

**Figure 2:**
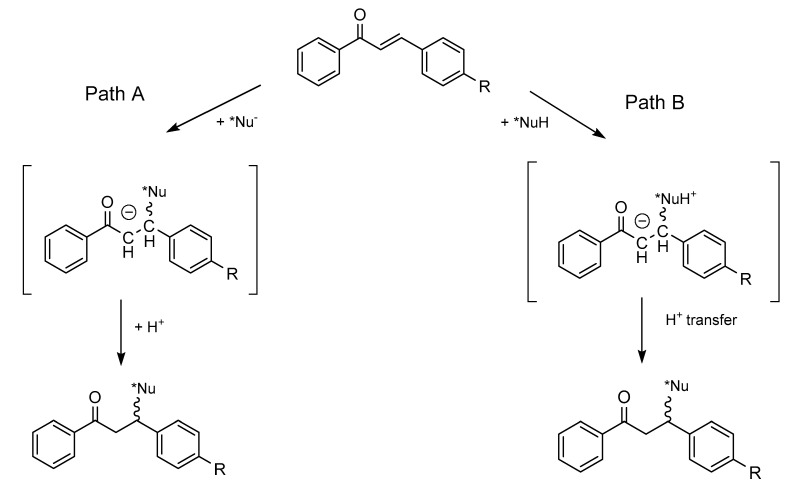
General mechanisms of the addition reaction of nucleophiles onto the activated double bonds of chalcones.

The Michael reaction refers to the addition of a nucleophile (Michael donor) to an activated α,β-unsaturated carbonyl compound (Michael acceptor), and it is typically base-catalyzed. Among the most commonly studied nucleophiles, one finds hydroxide ions (OH^−^), water, and amines; a more limited number of investigations have been reported with thiolate ions (ArS^−^, RS^−^), oxide ions (ArO^−^, RO^−^), and cyanide ions (CN^−^). The reactions can also be categorized based on the charge of the nucleophilic reactants, which could be (a) negatively charged (e.g., OH^−^, RS^−^, CN^−^) or (b) neutral (e.g., amines, thiols). Both kinds of nucleophiles can be added onto protonated or non-protonated α,β-unsaturated enones. Since the experimental p*K*_b_ value of (*E*)-chalcone was reported to be −5.00 [25], only additions onto the non-protonated chalcones were taken into consideration. The general mechanisms of the addition of deprotonated (path A) and neutral (path B) nucleophiles onto non-protonated chalcones are shown in Figure 2.

As shown in Figure 2, the primary adduct of the addition reactions either possesses a negative charge (path A) or has a zwitterion structure (path B). The second step of the reactions is an acid–base process, which can occur in an intermolecular or—in the case of the zwitterion intermediates—an intramolecular manner [26].

Previously, we reported the synthesis and reactions of two 4′-hydroxychalcones and their bis-methyleneamino (Mannich) derivatives. It was found that the Mannich derivatives showed significantly increased reactivity [27] and diastereoselectivity [28] in comparison to the respective non-Mannich derivatives when the reactions were conducted under acidic (about pH 3) conditions.

We investigated the reactions under three conditions with different pH: (a) pH 8.0/8.53, (b) pH 6.3/6.8, and (c) pH 3.2/3.7. The first pH values indicate the pH of the aqueous solution of the thiols before starting the incubations. The second pH values indicate the virtual pH of the incubation mixtures, which contained 75.5% *v/v* methanol (MeOH). The basic pH was selected because such conditions mimic the GST-catalyzed reactions; the ionization of the GSH thiol moiety increases due to its interaction with the basic imidazole N atom in the active site of the enzyme [29]. The slightly acidic conditions resemble the slightly acidic pH of the cancer cells [30]. The stronger acid conditions were selected to compare the reactivity of the protonated (neutral) and the ionized forms of the thiol functions of the two compounds. The p*K*_a_ of the thiol group of GSH and NAC was reported to be 8.83 and 9.52, respectively [18]. Accordingly, the thiol function of both compounds exists exclusively in the protonated (neutral) form under such acidic conditions. The stereoselectivity of the reactions was monitored by comparing the HPLC peak areas of the respective chalcone–GSH and chalcone–NAC adducts.

The reactions are reported to be reversible, accordingly resulting in the formation of an equilibrium mixture. To qualitatively characterize the progress of the addition processes, the composition of the incubation mixtures was analyzed at the 15, 45, 75, 105, 135, 165, 195, 225, 255, 285, and 315 min time points by HPLC-UV-VIS.

## 2. Results

### 2.1. Reactions under Basic (pH 8.0/8.5) Conditions

**Figure 3:**
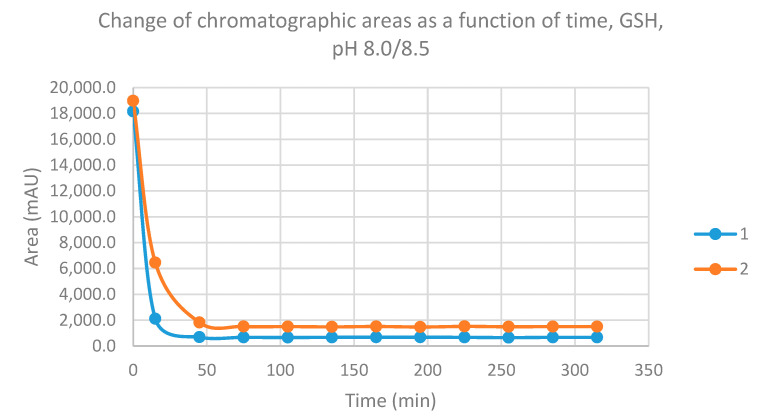
Change in the chromatographic peak area of chalcones **1** and **2** in the chalcone–GSH incubations at pH 8.0/8.5.

**Figure 4:**
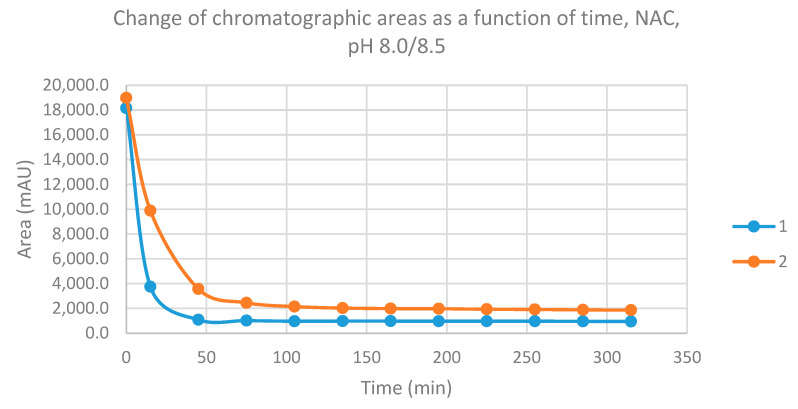
Change in the chromatographic peak area of chalcones **1** and **2** in the chalcone–NAC incubations at pH 8.0/8.5.

Initially, we investigated the reactions of the two chalcones under basic conditions. Considering the p*K*_a_ values of GSH (8.83) and NAC (9.52), about 31.9% of the GSH molecules and 8.7% of the NAC molecules existed under pH 8.5 conditions (the virtual pH of the incubates) in the more reactive thiolate form. By the end of the investigated period (315 min), the initial area of the HPLC peak corresponding to the parent compounds **1** and **2** reduced to 3.7% and 7.9%, respectively (Table 1). While the compounds were incubated with NAC, the respective figures were 5.2% and 9.8% (Table 2). Progress curve changes in the chromatographic peak areas of the starting chalcones (**1** and **2**) as a function of the incubation time of the reactions indicated that the compositions reflect those of the equilibrium in each case (Figure 3 and Figure 4).

As a result of the addition reactions, a new chiral center was formed. Considering the inherent chirality of the two thiols, the formation of two diastereomeric adducts was expected. However, using our HPLC conditions, the **1**-GSH and **2**-GSH conjugates were not separated (Table 1).

In the case of NAC incubations, the formed **1**-NAC and **2**-NAC adducts were only partially separated. Based on the integration of the two overlapping peaks, the ratio of the two diastereomeric adducts (NAC–**1** and NAC–**2**) showed a different (1.7–1.2 times) excess of the less polar diastereomers (Table 2). The structure of the parent chalcones (**1** and **2**) as well as their GSH and NAC conjugates was verified by HPLC-MS (Table S1 and Figures S10–S15).

**Figure 5:**
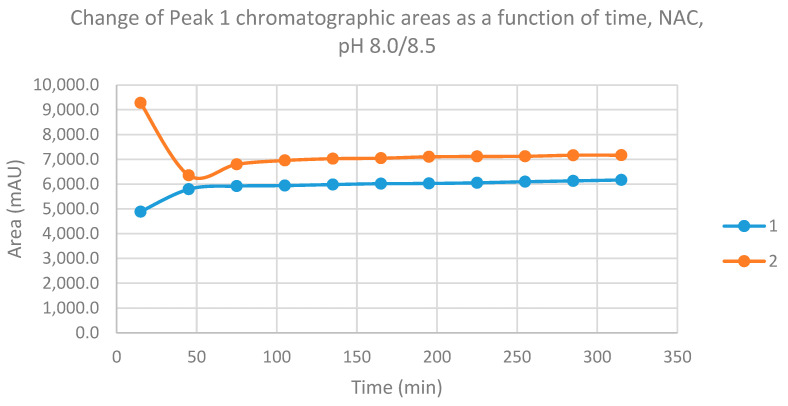
Change in the chromatographic peak area of adduct 1 of chalcones **1** and **2** in the chalcone–NAC incubations at pH 8.0/8.5.

**Figure 6:**
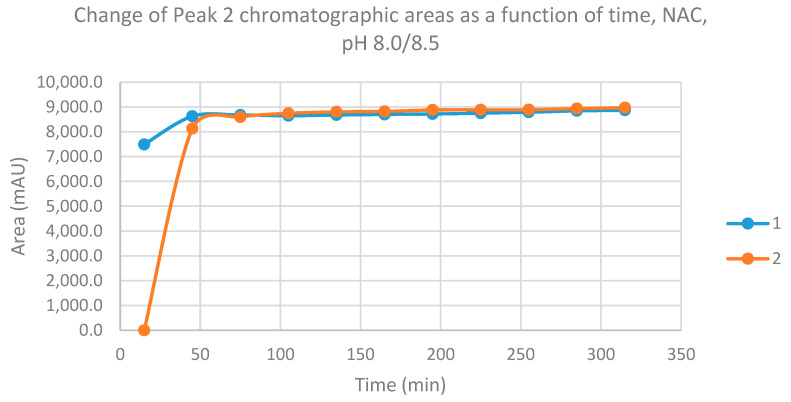
Change in the chromatographic peak area of adduct 2 of chalcones **1** and **2** in the chalcone–NAC incubations at pH 8.0/8.5.

The progress curves of the formation of the diastereomeric NAC adducts—based on the integrated HPLC peak areas (AUCs)—are shown in Figure 5 and Figure 6. As shown, the formation of the **1**-NAC diastereomers is increased in the first 45 min and remained the same over the time of incubation. In the case of the **2**-NAC diastereomers, rapid formation of the kinetic product (NAC–**1**) was observed in the first 15 min. After that, however, the NAC–**1** isomer of **2** was transisomerized to the thermodynamic product (NAC–**2**), reaching the equilibrium composition by the 105 min time point (Figure 5 and Figure 6).

Further to the above data, it is worth mentioning that during the incubations with GSH and NAC, small new peaks appeared in the chromatograms with a somewhat shorter retention time than that of unreacted **1** and **2** (Table 1 and Table 2). Based on our previous results [31], the new peaks were supposed to be those of the respective (*Z*) diastereomers. Since such isomerization could not be observed in the incubations performed without the thiols, the formation of the (*Z*) isomer can be considered due to the retro-Michael reaction. To identify the structure of the expected (*Z*) diastereomers, light-initiated isomerization of **1** and **2** was performed. Based on the result of the light-initiated isomerization experiment, the formed compounds were identified as the respective (*Z*) isomers (Figures S16 and S17).

### 2.2. Reactions under Slightly Acidic (pH 6.3/6.8) Conditions

Reactions under slightly acidic conditions mimic the cellular milieu of cancer cells [30]. Under these experimental conditions (pH 6.8), about 0.9% of GSH and 0.2% of NAC molecules exist in the more reactive thiolate form. The progress of the reactions under such conditions was more restricted than that observed at pH 8.0/8.5. In the GSH incubations, the initial area of the parent compounds **1** and **2** was reduced to 9.4% and 21.4%, respectively, by the end of the investigated period (Table 1). The respective figures for the NAC incubations were 24.4% and 46.8% (Table 2).

**Figure 7:**
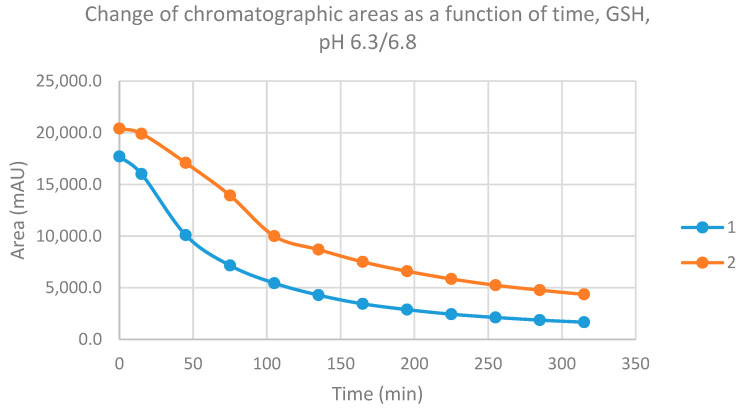
Change in the chromatographic peak area of chalcones **1** and **2** in the chalcone–GSH incubations at pH 6.3/6.8.

**Figure 8:**
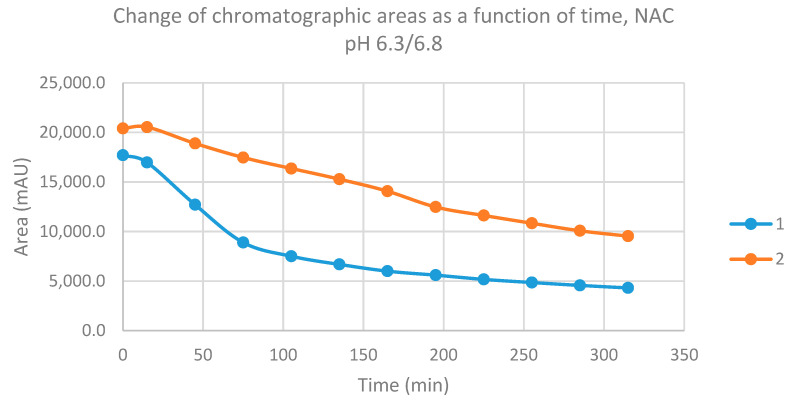
Change in the chromatographic peak area of chalcones **1** and **2** in the chalcone–NAC incubations at pH 6.3/6.8.

Progression curves of the reactions (Figure 7 and Figure 8) indicated that the percentage figures represent compositions close to equilibrium. Similar to the results obtained under pH 8.0/8.5 conditions, the formation of a small amount of (*Z*) isomers was detected in the incubation mixtures.

Progression curves of the formation of the chalcone–GSH and chalcone–NAC adducts showed two parallel concave curves with finite limits (Figures S1–S4).

### 2.3. Reactions under Acidic (pH 3.2/3.7) Conditions

Reactions under stronger acidic conditions proceeded to a much lower extent than those under the above two conditions. Under stronger acidic conditions, the thiol function of both GSH and NAC exists exclusively in protonated (neutral) form. Although protonated thiols can act as nucleophilic reagents, their reactivity is much lower than that of their deprotonated (negatively charged) counterparts [32].

Only a small amount of adducts were detected in each case in the chalcone–GSH/NAC incubates (Table 2). The chromatographic peak area values of the (*Z*) isomers were similar to those in the respective incubates at pH 8.0/8.5 and pH 6.3/6.8 (Table 1 and Table 2).

Progression curves of the reaction of chalcones with GSH showed a linear downhill shape (Figure S5). A similar linear reduction in the chromatographic peak areas was observed in the NAC incubations (Figure S6).

Over the whole incubation period, the chromatographic peak areas of the chalcone–GSH (Figure S7) and chalcone–NAC diastereomers continuously increased (Figures S8 and S9).

## 3. Discussion

Our experiments demonstrated that both GSH and NAC react with the investigated chalcones under acidic (pH 3.2/3.7, pH 6.3/6.8) and basic (pH 8.0/8.5) conditions. However, the rate of the initial reactions and the composition of the equilibrium mixtures were affected by the nature of the reactants and the pH of the incubation mixtures.

Analysis of the effect of the 4-substituents under basic (pH 8.0/8.5) or slightly acidic (pH 6.3/6.8) conditions showed 4-methyl-substituted **1** to display higher initial reactivity. ^13^C NMR shifts, indicating the electron density around the particular nucleus of the *beta*-C atom of **1** (144.9 ppm) and **2** (144.6 ppm), were reported to be similar [33]. The observed difference in the reactivity of chalcones **1** and **2** can be explained by the stability of the thiol adducts. An early work of Humphlett et al. demonstrated that the activity of the α-hydrogen atom of the adduct, the resonance stabilization of the enone formed by cleavage, and the anionic stability of the thiolate ion are the determining factors of the reverse process. The authors found the α-keto and β-phenyl substitutions as determining factors in the effective reverse reactions [34]. Since the 4-methoxy substitution can more effectively increase the electron density on the carbon–carbon double bond, and the formed chalcone is resonance stabilized, the elimination process is more effective in the case of **2** than **1**. Similar conclusions were made by d’Oliveira et al. while investigating a few chalcones and their rigid quinolone analogues [35].

The retro-thia-Michael reactions can result in the formation of the respective (*Z*) isomers as well. Therefore, to obtain authentic reference (*Z*) isomers, the stereochemically homogeneous (*E*) isomers (**1** and **2**) were submitted with light-initiated isomerization, as published before [30]. As a result, HPLC-MS data agreed with the respective (*Z*) isomers (Figures S16 and S17).

Comparison of the p*K*_a_ values of GSH (8.83) and NAC (9.52) thiols, and the respective conversions of the starting chalcones (Table 1 and Table 2), showed that the higher the p*K*_a_ of the thiol, the lower the conversion of the chalcones in the case of both derivatives at each investigated pH. This observation reflects the importance of acidity (*K*_a_) of the thiol group that regulates the relative amount of RS^−^ concerning RSH, hence the equilibrium.

HPLC analysis of the reactions of **1** and **2** with NAC showed a different (1.7–1.2 times) excess of the less polar diastereomer (Table 2). The observed diastereoselectivity was affected by both the nature of the 4-substituent and the pH. Thus, at each different pH value, it was the methyl-substituted **1** that showed higher diastereoselectivity. In contrast, in the case of both chalcones, diastereoselectivity decreased with the increase in the pH. HPLC-UV-Vis chromatograms of the chalcone **1**-NAC incubations at pH 8.0/8.3, pH 6.3/6.8, and pH 3.2/3.7 (315 min time point) are shown in Figures S18–S20, respectively. The corresponding HPLC-UV-Vis chromatograms of the chalcone **2**-NAC incubations are shown in Figures S21–S23.

**Figure 9:**
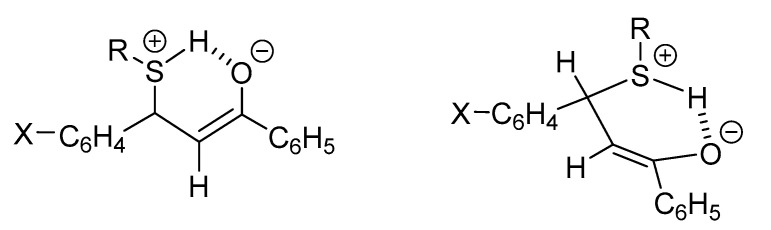
Possible enolate intermediate of the addition of protonated thiols onto chalcones.

These observations give additional indirect support to the formation of a six-membered, hydrogen-bond-stabilized cyclic intermediate in the reactions of chalcones with the protonated form of thiols. The formation of such a cyclic intermediate was suggested earlier in the reaction of GSH with the bis-Mannich derivatives of 4′-hydroxychalcones [27]. According to our previous explanation, the protonated thiol attacks the planar enone moiety from the *Re*-side, resulting in a six-membered intermediate with a pseudoequatorial position of the bulky aryl ring (Figure 9).

Michael-type thiol reactivity of chalcones and related compounds is frequently associated with biological activities [6,7,8,9,10,11,36]. In contrast, several examples demonstrate that non-covalent interactions of chalcones with cellular macromolecules can play an important role in the biological effects of the compounds [37]. In a QSAR study, Katsori et al. found the clog *P* parameter to play an important part in the QSAR relationships. The authors found the electronic effects are comparatively unimportant in the anticancer effect of the investigated chalcones [38]. Comparison of the spontaneous thiol reactivities of the two chalcones under pH 8.0/8.5 and pH 6.3/6.8 conditions toward GSH and NAC showed some characteristic differences. Conversion of **1** with both GSH and NAC was higher than **2** under all investigated conditions (Table 1 and Table 2). The experimental log *P* of **2** and **3** has been reported to be 4.12 and 3.67, respectively [39]. Thus, it is reasonable to suppose that both the thiol reactivity and the lipophilic properties contributed to the reported biological effects of the investigated compounds and their cyclic analogues.

## 4. Materials and Methods

### 4.1. Chemicals and Reagents

Chalcones **1** and **2** were synthesized, as reported before [40]. Their purity was tested by TLC and HPLC-UV-VIS. Reduced l-glutathione and *N*-acetyl l-cysteine were obtained from Sigma-Aldrich (Budapest, Hungary). The methanol CHROMASOLV gradient for HPLC was obtained from Honeywell (Hungary). Trifluoroacetic acid HiperSolve CHROMANORM was obtained from VWR (Budapest, Hungary) and formic acid from Fischer Chemicals. Deionized water for use in HPLC and HPLC-MS measurements was purified by Millipore Direct-Q^TM^ at the Institute of Pharmaceutical Chemistry (University of Pécs). Mobile phases used for HPLC measurements were degassed by an ultrasonic water bath before use.

### 4.2. Preparation of Solutions

To evaluate the reactivity of the investigated chalcones and their analogues with thiols, reduced glutathione (GSH) and *N*-acetylcysteine (NAC) solutions were prepared as follows: Each solution was prepared at three different pH values (3.2, 6.3, and 8.0). The pH was set using 1M NaOH solution. (a) Solutions of GSH and NAC were prepared in water to a final volume of 1.5 cm^3^ with a concentration of 2.0 × 10^−1^ mol·L^−1^ (0.3 mmol thiol). (b) Chalcone solutions were freshly prepared before incubation to a 4.6 cm^3^ volume of HPLC-grade methanol with a concentration of 6.5 × 10^−3^ mol·L^−1^ (0.03 mmol chalcone). (c) The GSH/NAC and chalcone solutions were pre-incubated in a 37 °C water bath for 15 min in the dark. Then, the solutions were mixed, resulting in a mixture of the thiol and the chalcone in a molar ratio of 10:1. The mixture was kept in the dark in a temperature-controlled (37 °C) water bath for a total duration of 315 min. To monitor the reaction by RP-HPLC, samples were taken at time points of 15, 45, 75, 105, 135, 165, 195, 225, 255, 285, and 315 min.

To evaluate the initial (0 min) peak area of chalcones **1** and **2**, 4.6 cm^3^ methanolic solution of each was prepared as above (method (b)), and the solutions were diluted with 1.5 mL of aqueous solution with the respective pH before analysis. Before mixing, the solutions were pre-incubated at 37 °C for 30 min.

To compare the products of the previously proven light-initiated *E/Z* isomerization of the parent compounds [26] with those of the non-light (retro-Michael addition)-initiated isomerization, solutions of chalcones **1** and **2** were prepared by method (b), and the solutions were subjected to unscattered laboratory light for 1 week. The solutions were analyzed by HPLC-UV-VIS and HPLC-MS.

### 4.3. RP-HPLC-UV-VIS Measurements

The measurements were performed on an Agilent 1100 HPLC system coupled with a UV–VIS detector. The wavelength was set at 260 nm. The separation of the components was carried out in a reversed-phase chromatographic system. A Zobrax Eclipse XBD-C8 column (150 mm × 4.6 mm, particle size 5 µm; Agilent Technologies, Waldbronn, Germany) was used. The injection volume was 10 µL. During the time of the measurement, the column oven was set at room temperature (25 °C). Data were recorded and evaluated by the use of Agilent Chem Station (B.03.01). Gradient elution was performed at a flow rate of 1.2 mL/min; the mobile phase consisted of (A) water and 0.1% trifluoroacetic acid and (B) methanol and 0.1% trifluoroacetic acid. The gradient profile was as follows: an isocratic period of 8 min of 40% mobile phase B, followed by a linear increase to 60% in 4 min, a second linear gradient to 90% in 3 min, and a 5 min isocratic period of 90%. The column was then equilibrated to the initial conditions with a 2 min linear gradient to 40%, followed by 3 min of the isocratic period.

### 4.4. HPLC-MS Measurements

HPLC ESI-MS analyses were performed on an Ultimate 3000 liquid chromatograph (Dionex, Sunnyvale, CA, USA) coupled with a Thermo Q Exactive Focus quadrupole-Orbitrap hybrid mass spectrometer (Thermo Fisher Scientific, Waltham, MA, USA). The scan monitored *m/z* values ranging from 100 to 1000 Da. Data acquisition was carried out using Q Exactive Focus 2.1 and Xcalibur 4.2 software (Thermo Fisher Scientific). Analysis of compounds and adducts was performed in HESI positive and negative ionization modes with the following parameters: spray voltage, 3500 V; vaporizer temperature, 300 °C; capillary temperature, 350 °C; spray and auxiliary gas flows, 30 and 10 arbitrary units, respectively; resolution, 35,000 at 200 *m/z*; and fragmentation, 20 eV.

HPLC separation was performed on an Accucore C18 column (150 mm × 2.1 mm, particle size 2.6 µm), and an Accucore C18 guard column (5 mm × 2.1 mm, particle size 2.6 µm) was also used. The injection volume was 5 µL; the flow rate was set to 0.4 mL/min. Data analysis and evaluations were performed using Xcalibur 4.2 and FreeStyle 1.7 software. A binary gradient of eluents was used, consisting of mobile phases A and B.

The parameters of the gradient in chalcones were (A) water and 0.1% formic acid and (B) methanol and 0.1% formic acid. The gradient elution was as follows: isocratic elution for 1 min to 20% eluent B, continued by a linear gradient to 100% in 9 min, followed by an isocratic plateau for 2 min. Then, the column was equilibrated back to 20% in 0.5 min and continued isocratically for 2.5 min. The sampler was at room temperature, and the column oven was kept at 40 °C.

The parameters of the gradient in the case of adducts were (A) water and 0.1% formic acid and (B) methanol and 0.1% formic acid. The gradient elution was as follows: isocratic elution for 1 min to 10% eluent B, continued by a linear gradient to 95% in 13 min, followed by an isocratic plateau for 3 min. Finally, the column was equilibrated back to 10% in 0.1 min and continued isocratically for 2.9 min. The sampler was at room temperature, and the column oven was kept at 40 °C. The diode array detector was also set at 260 nm wavelength alongside MS analysis.

## 5. Conclusions

The present work aimed to investigate the thiol reactivity of two 4-substituted chalcone derivatives (**1** and **2**) with different experimental log-*P*-values. HPLC-UV-VIS and HPLC-MS investigations of spontaneous reactions of the two chalcones at three different pH values revealed that the compounds have similar reactivities. Furthermore, comparison of the composition of the equilibrium mixtures revealed the importance of the electronic effect of the 4-substituent on the stability of the enolate intermediates.

HPLC analysis of the incubation mixtures of **1** and **2** with NAC demonstrated different stereochemistry of the addition of the protonated and non-protonated thiol nucleophile onto the enone moiety. The results provided further support for the dominant conformation-derived diastereoselective addition of the protonated thiols onto the chalcones’ polar carbon–carbon double bond.

We could not find a direct correlation of the thiol reactivities and the previously published biological (cancer cell cytotoxic) effects of chalcones **1** and **2**. It is reasonable to suppose that both thiol reactivities and lipophilic properties contributed to the reported biological effects of the investigated compounds and their cyclic analogues.

## Figures and Tables

**Table 1:** Retention times (t_R_) ^1^ and integrated peak areas (A) of the investigated chalcones (**1** and **2**) and their GSH adducts ^2^.

pH ^3^	Compound	t_R_ (*E*)-Chalcone	Area Ratio ^4^ A_315_/A_0_	t_R_ (*Z*)-Chalcone	Area (*Z*)-Chalcone	t_R_ GSH–1	Area GSH–1	t_R_ GSH–2	Area GSH–2
3.2	1	16.4	0.81	16.2	<100	13.8	4245	N/D ^5^	-
3.2	2	15.9	0.96	15.7	<100	11.9	3352	N/D ^5^	-
6.3	1	16.3	0.09	16.0	<100	13.2	16,571	N/D ^5^	-
6.3	2	15.8	0.21	15.5	<100	11.3	17,160	N/D ^5^	-
8	1	16.3	0.04	16.1 ^6^	<100	13.3	17,419	N/D ^5^	-
8	2	15.7	0.08	15.5	<100	11.0	20,387	N/D ^5^	-

^1^ Retention times in minutes; ^2^ data refer to the average of two independent measurements at the 315 min time point; ^3^ pH value of the aqueous thiol solution; ^4^ ratio of peak areas measured at 0 and 315 min; ^5^ not detectable; ^6^ only detectable at the 15, 45, 75, 135, and 165 min time points.

**Table 2:** Retention times (tr) ^1^ and integrated peak areas (A) of the investigated chalcones (**1** and **2**) and their NAC adducts ^2^.

pH ^3^	Compound	t_R_ (*E*)-Chalcone	Area Ratio ^4^ A_315_/A_0_	t_R_ (*Z*)-Chalcone	Area (*Z*)-Chalcone	t_R_ NAC–1	Area NAC–1	t_R_ NAC–2	Area NAC–2
3.2	1	16.3	0.89	16.1	<100	15.2	1260	15.3	2173
3.2	2	15.8	0.98	15.5	<100	14.1	1156	14.2	1507
6.3	1	16.3	0.24	16.0	<100	15.1	4906	15.2	6457
6.3	2	15.8	0.47	15.5	<100	14.1	4712	14.2	5422
8	1	16.2	0.05	16.0	<100	15.1	6167	15.2	8875
8	2	15.7	0.10	15.5	<100	14.1	7167	14.2	8975

^1^ Retention times in minutes; ^2^ data refer to the average of two independent measurements at the 315 min time point; ^3^ pH value of the aqueous thiol solution; ^4^ ratio of peak areas measured at 0 and 315 min.

## Data Availability

Not applicable.

## Supplementary Materials

The following are available: Table S1. Mass spectrometry data; Figure S1. Change in the chromatographic peak area of adduct 1 of chalcones **1** and **2** in the chalcone–GSH incubations at pH 8.0/8.5. Figure S2. Change in the chromatographic peak area of adduct 1 of chalcones **1** and **2** in the chalcone–GSH incubations at pH 6.3/6.8. Figure S3. Change in the chromatographic peak area of adduct 1 of chalcones **1** and **2** in the chalcone–NAC incubations at pH 6.3/6.8. Figure S4. Change in the chromatographic peak area of adduct 2 of chalcones **1** and **2** in the chalcone–NAC incubations at pH 6.3/6.8. Figure S5. Change in the chromatographic peak area of chalcones **1** and **2** in the chalcone–GSH incubations at pH 3.2/3.7. Figure S6. Change in the chromatographic peak area of chalcones **1** and **2** in the chalcone–NAC incubations at pH 3.2/3.7. Figure S7. Change in the chromatographic peak area of adduct 1 of chalcones **1** and **2** in the chalcone–GSH incubations at pH 3.2/3.7. Figure S8. Change in the chromatographic peak area of adduct 1 of chalcones **1** and **2** in the chalcone–NAC incubations at pH 3.2/3.7. Figure S9. Change in the chromatographic peak area of adduct 2 of chalcones **1** and **2** in the chalcone–NAC incubations at pH 3.2/3.7. Figure S10. High-resolution, positive-mode HESI MS spectrum of chalcone **1**. Figure S11. High-resolution, positive-mode HESI MS spectrum of chalcone **2**. Figure S12. High-resolution, positive-mode HESI MS spectrum of chalcone **1**–GSH conjugate. Figure S13. High-resolution, negative-mode HESI MS spectrum of chalcone **1**–NAC conjugate. Figure S14. High-resolution, positive-mode HESI MS spectrum of chalcone **2**–GSH conjugate. Figure S15. High-resolution, negative-mode HESI MS spectrum of chalcone **2**–NAC conjugate. Figure S16. HPLC-UV-VIS spectrum of (*E*)(t_R_ 16.354 min)/(*Z*)(t_r_ 16.128 min) isomeric mixture of chalcone **1**. Figure S17. HPLC-UV-VIS spectrum of (*E*)(t_R_ 15.607 min)/(*Z*)(t_r_ 15.855 min) isomeric mixture of chalcone **2**. Figure S18. HPLC-UV-VIS chromatogram of pH 8.0/8.5 incubation (315 min time point) of chalcone **1** and NAC. Figure S19. HPLC-UV-VIS chromatogram of pH 6.3/6.8 incubation (315 min time point) of chalcone **1** and NAC. Figure S20. HPLC-UV-VIS chromatogram of pH 3.2/3.7 incubation (315 min time point) of chalcone **1** and NAC. Figure S21. HPLC-UV-VIS chromatogram of pH 8.0/8.5 incubation (315 min time point) of chalcone **2** and NAC. Figure S22. HPLC-UV-VIS chromatogram of pH 6.3/6.8 incubation (315 min time point) of chalcone **2** and NAC. Figure S23. HPLC-UV-VIS chromatogram of pH 3.2/3.7 incubation (315 min time point) of chalcone **2** and NAC.

## Sample Availability

Samples of the compounds **1** and **2** are available from the authors.
