# Supplementary material for: Reaction of Chalcones with Cellular Thiols. The Effect of the 4-Substitution of Chalcones and Protonation State of the Thiols on the Addition Process. Diastereoselective Thiol Addition
Source: Molecules. 2021 Jul 17;26(14):4332. doi: 10.3390/molecules26144332 (PMC8308006; doi:10.3390/molecules26144332)

Supplementary materials

Figure S1. Change in the chromatographic peak area of Adduct-1 of chalcones **1** and **2** in the chalcone-GSH incubations of pH 8.0/8.5.

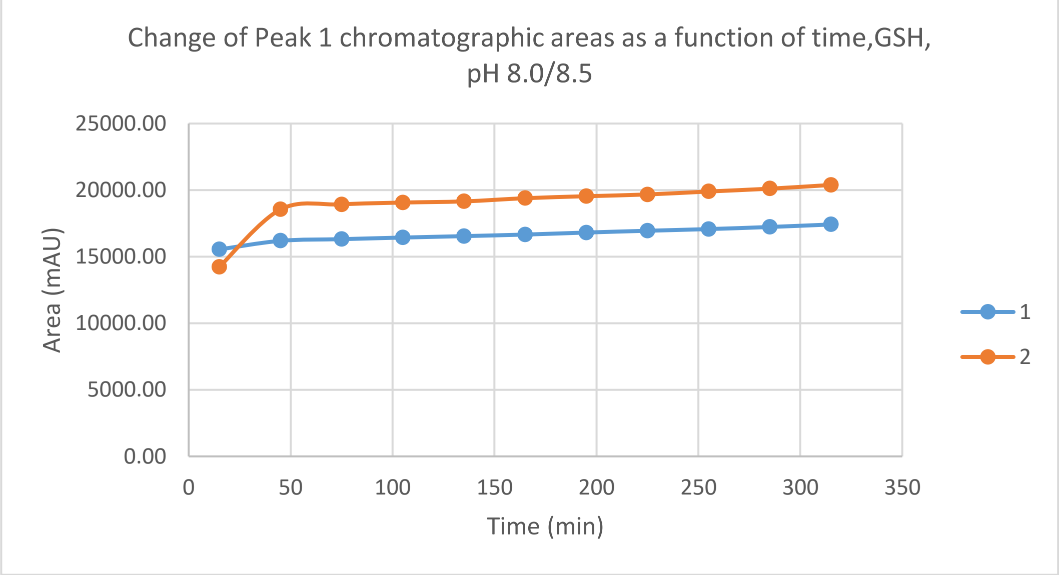

Figure S2. Change in the chromatographic peak area of Adduct-1 of chalcones **1** and **2** in the chalcone-GSH incubations of pH 6.3/6.8.

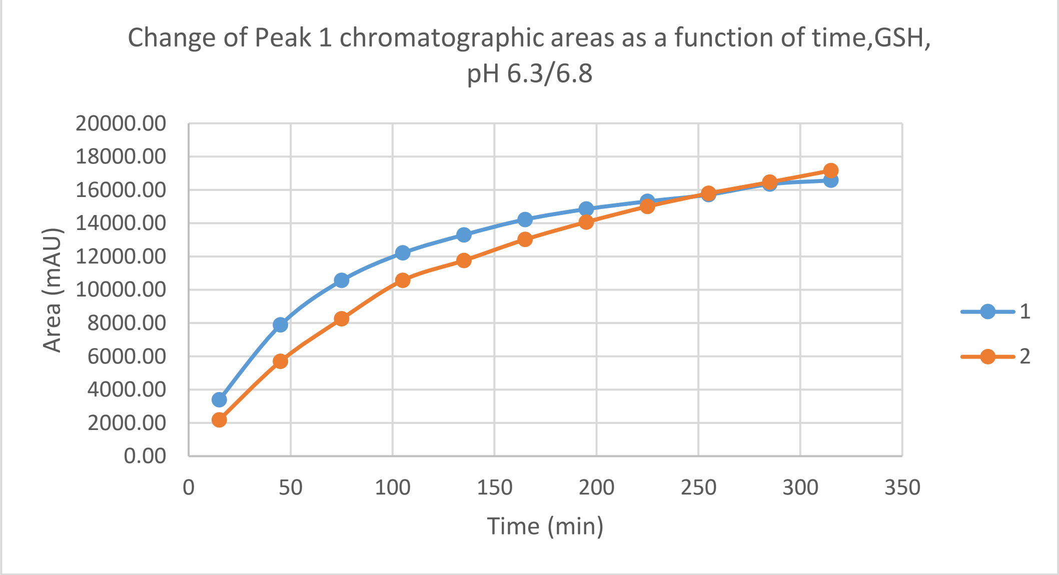

Figure S3. Change in the chromatographic peak area of Adduct-1 of chalcones **1** and **2** in the chalcone-NAC incubations of pH 6.3/6.8.

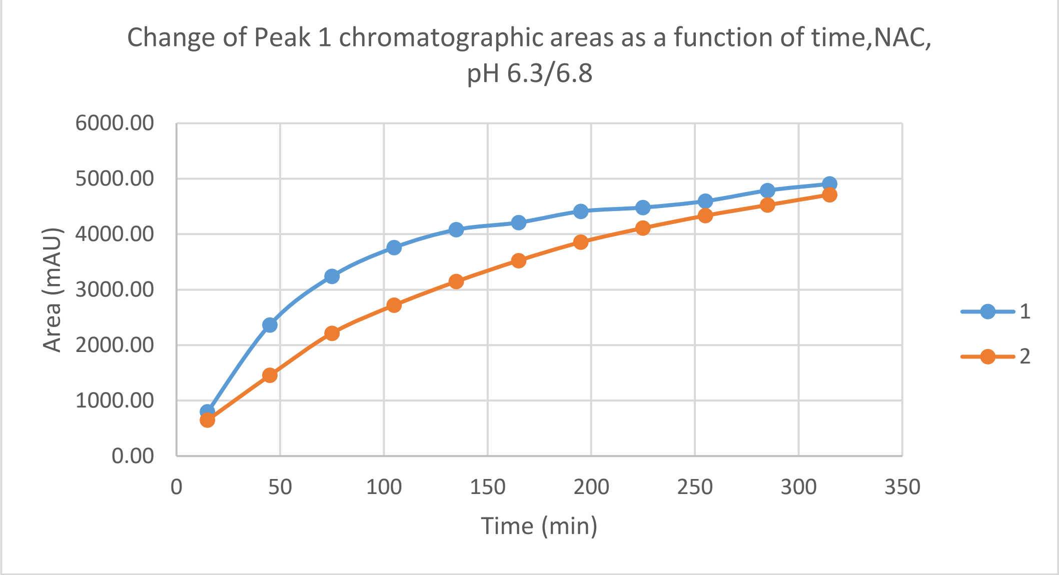

Figure S4. Change in the chromatographic peak area of Adduct-2 of chalcones **1** and **2** in the chalcone-NAC incubations of pH 6.3/6.8.

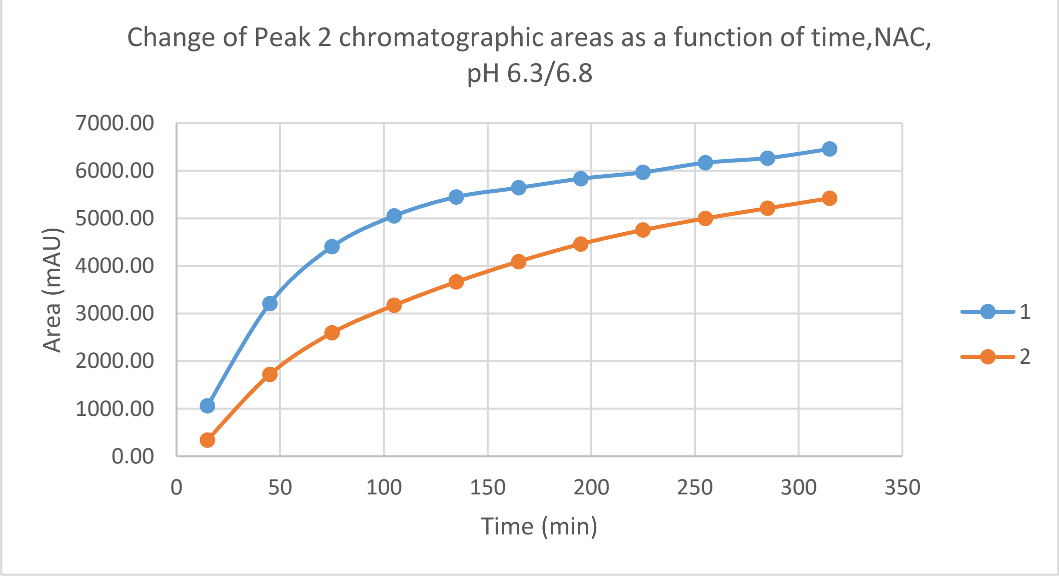

Figure S5. Change in the chromatographic peak area of chalcones **1** and **2** in the chalcone-GSH incubations of pH 3.2/3.7.

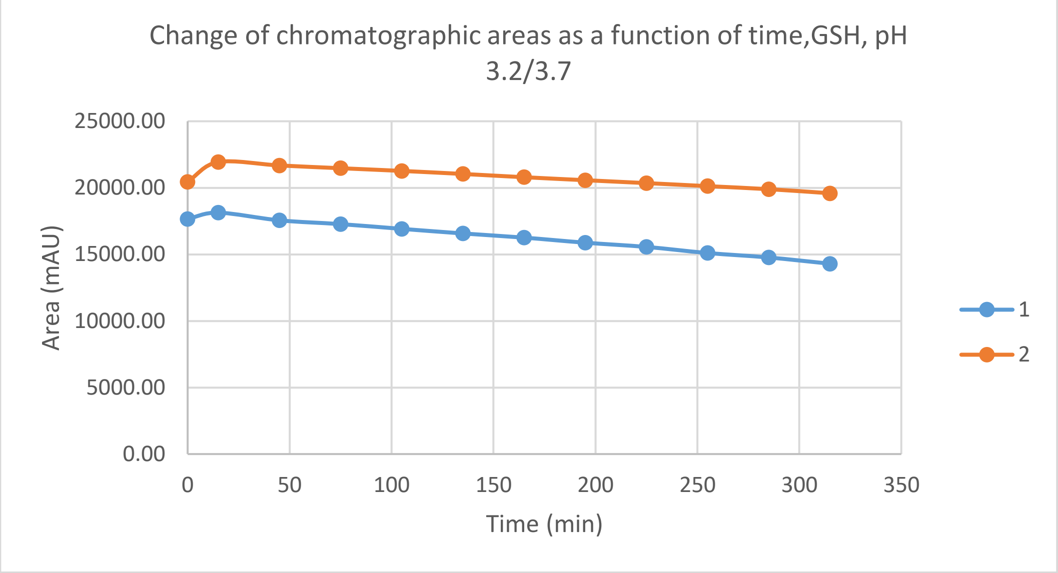

Figure S6. Change in the chromatographic peak area of chalcones **1** and **2** in the chalcone-NAC incubations of pH 3.2/3.7.

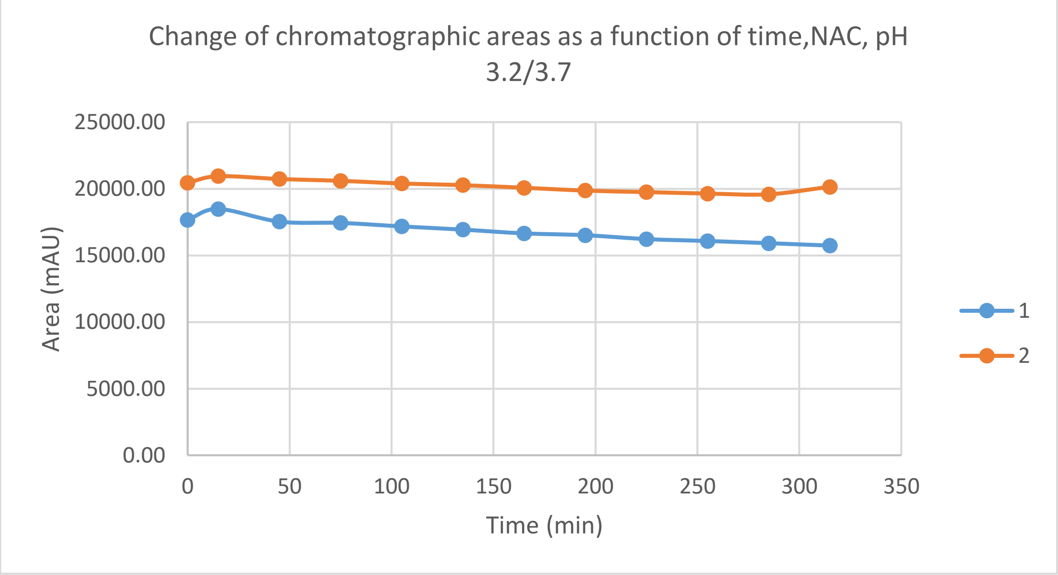

Figure S7. Change in the chromatographic peak area of Adduct-1 of chalcones **1** and **2** in the chalcone-GSH incubations of pH 3.2/3.7.

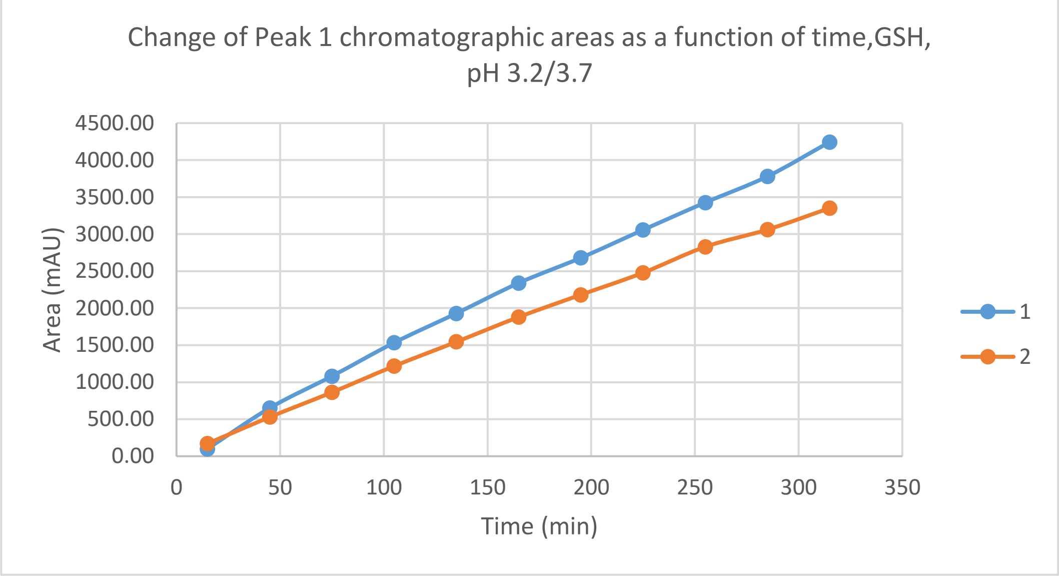

Figure S8. Change in the chromatographic peak area of Adduct-1 of chalcones **1** and **2** in the chalcone-NAC incubations of pH 3.2/3.7.

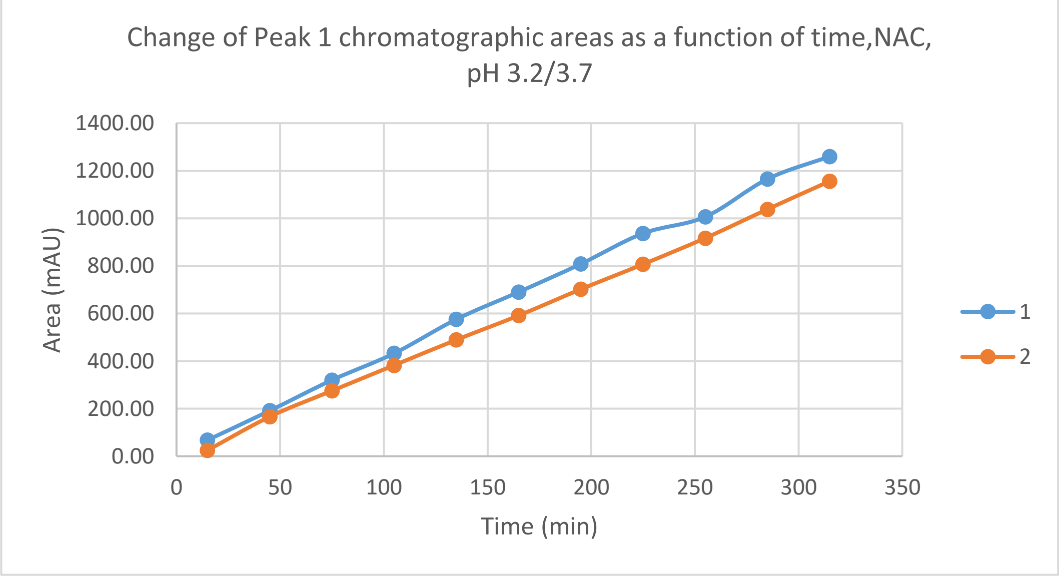

Figure S9. Change in the chromatographic peak area of Adduct-2 of chalcones **1** and **2** in the chalcone-NAC incubations of pH 3.2/3.

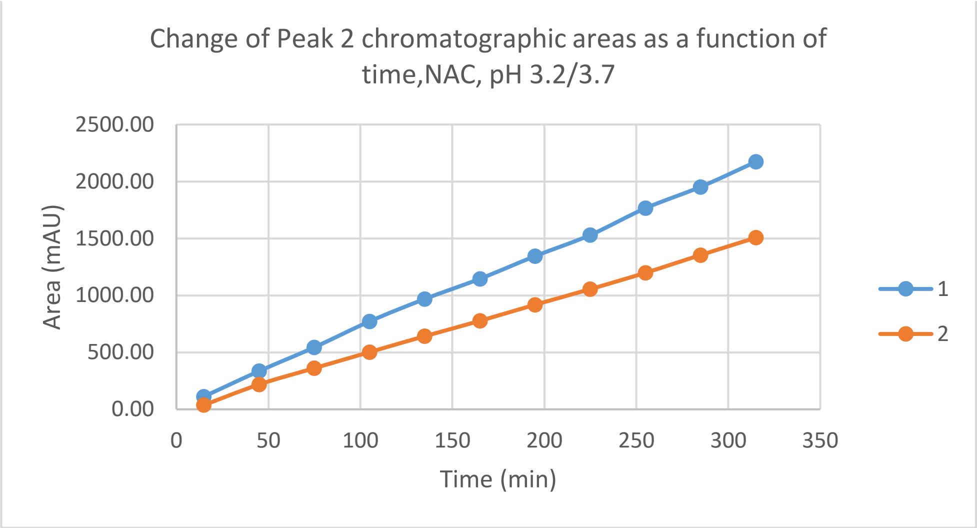

Table S1. Mass spectrometry data

Type	Value	**1**	**2**
Chalcone	Formula	$C_{16}H_{14}O$	$C_{16}H_{14}O_2$
	$[M+H]^+_{th}$	223.1123	239.1072
	$[M+H]^+_{m}$	223.1117	239.1066
	ΔM	-2.69	-2.51
Chalcone-GSH adduct	Formula	$C_{26}H_{31}N_3O_7S$	$C_{26}H_{31}N_3O_8S$
	$[M+H]^+_{th}$	530.1961	546.1910
	$[M+H]^+_{m}$	530.1946	546.1892
	ΔM	-2.83	-3.30
	Number of isomers	1	1
Chalcone-NAC adduct	Formula	$C_{21}H_{23}NO_4S$	$C_{21}H_{23}NO_5S$
	$[M-H]^-_{th}$	384.1270	400.1219
	$[M-H]^-_{m}$	384.1246	400.1191
	ΔM	-6.25	-7.00
	Number of isomers	2	2

Figure S10. High resolution, positive mode HESI MS spectrum of chalcone **1**.

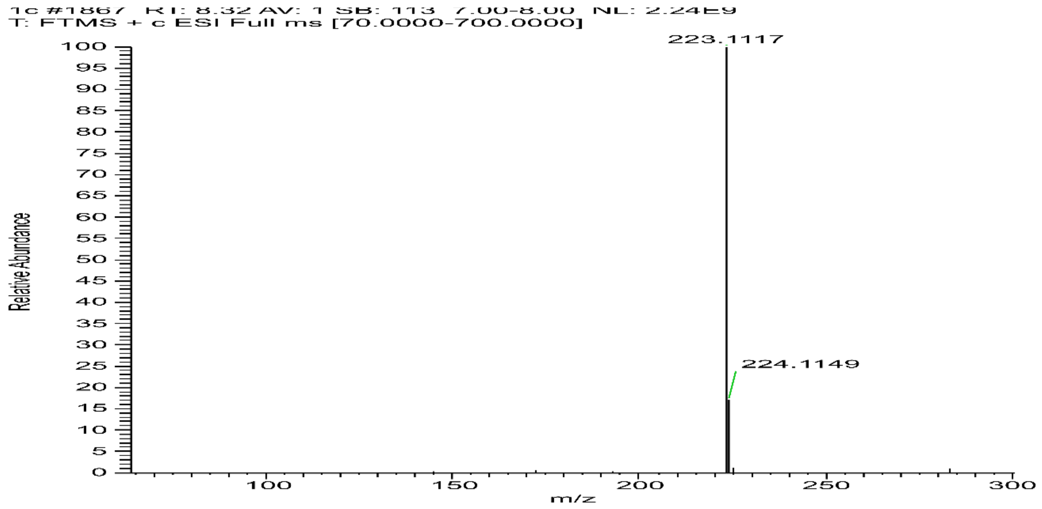

Figure S11. High resolution, positive mode HESI MS spectrum of chalcone **2**.

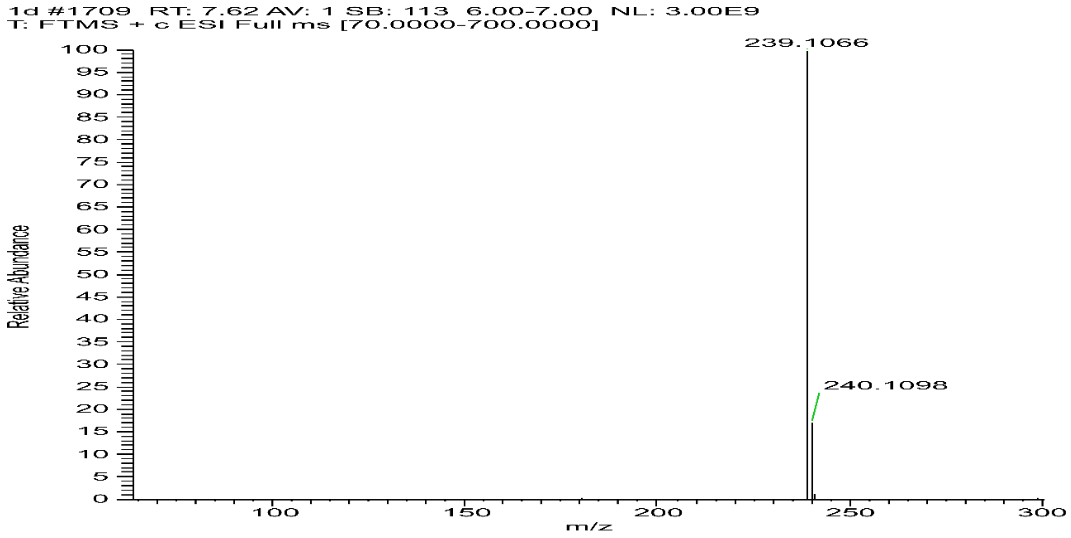

Figure S12. High resolution, positive mode HESI MS spectrum of chalcone **1**-GSH conjugate.

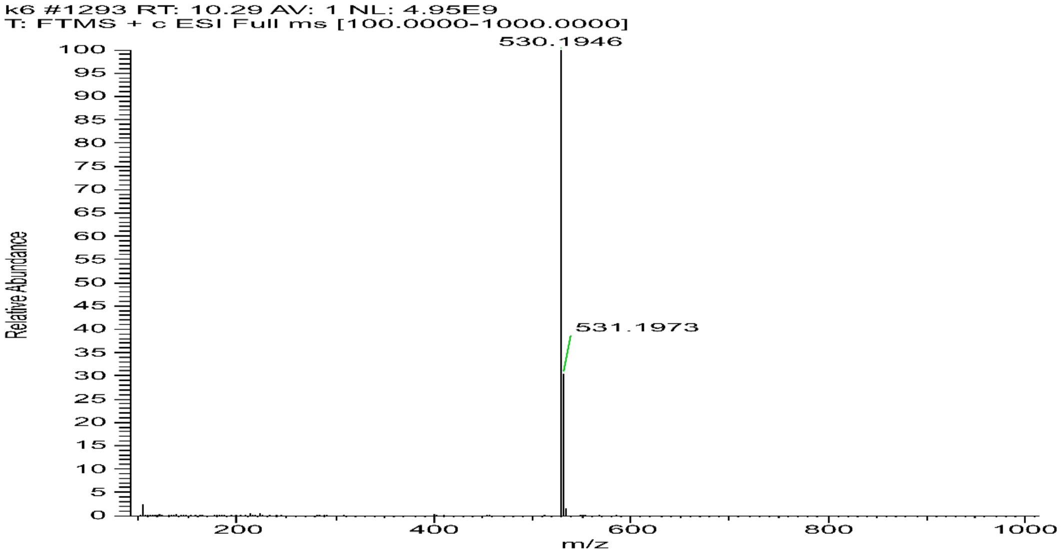

Figure S13. High resolution, negative mode HESI MS spectrum of chalcone **1**-NAC conjugate.

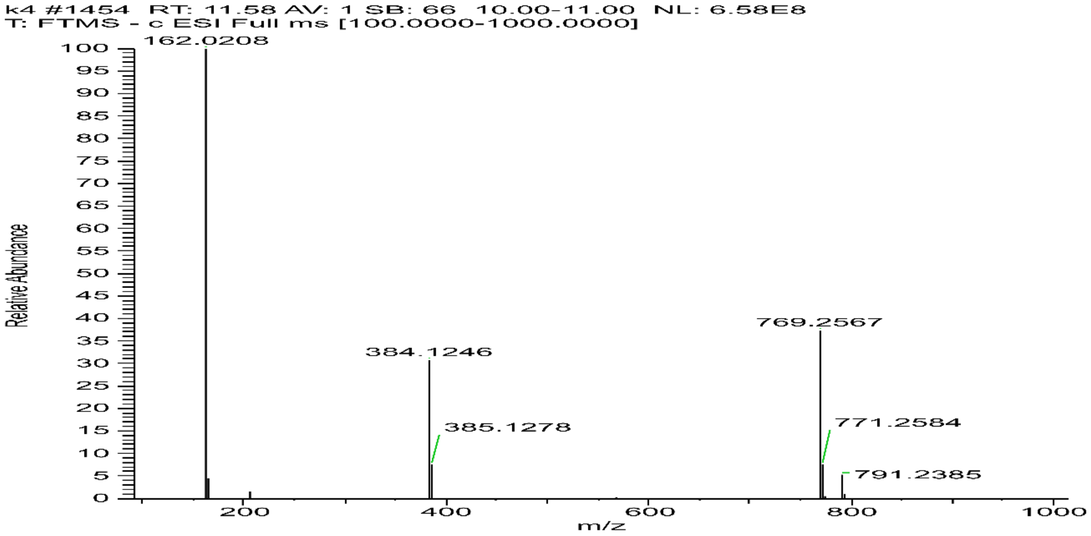

Figure S14. High resolution, positive mode HESI MS spectrum of chalcone **2**-GSH conjugate.

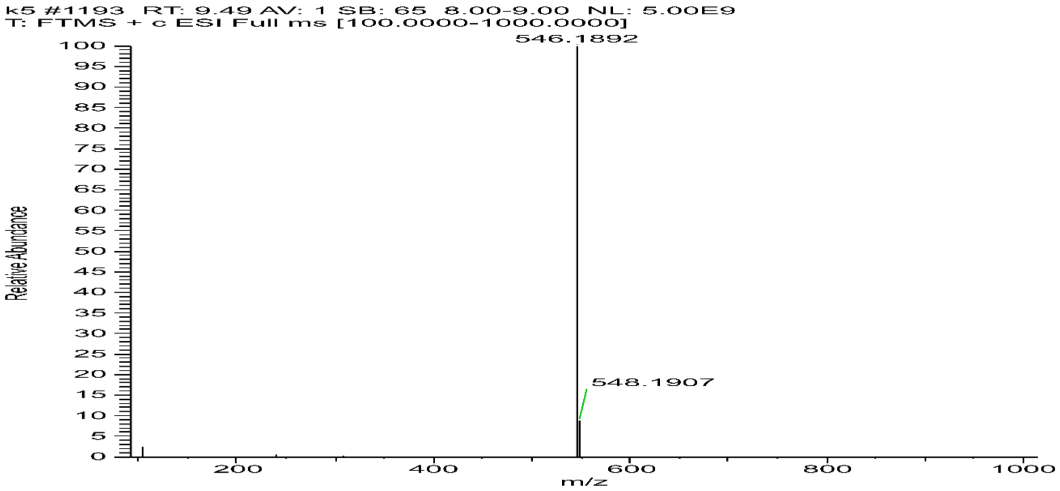

Figure S15. High resolution, negative mode HESI MS spectrum of chalcone **2**-NAC conjugate.

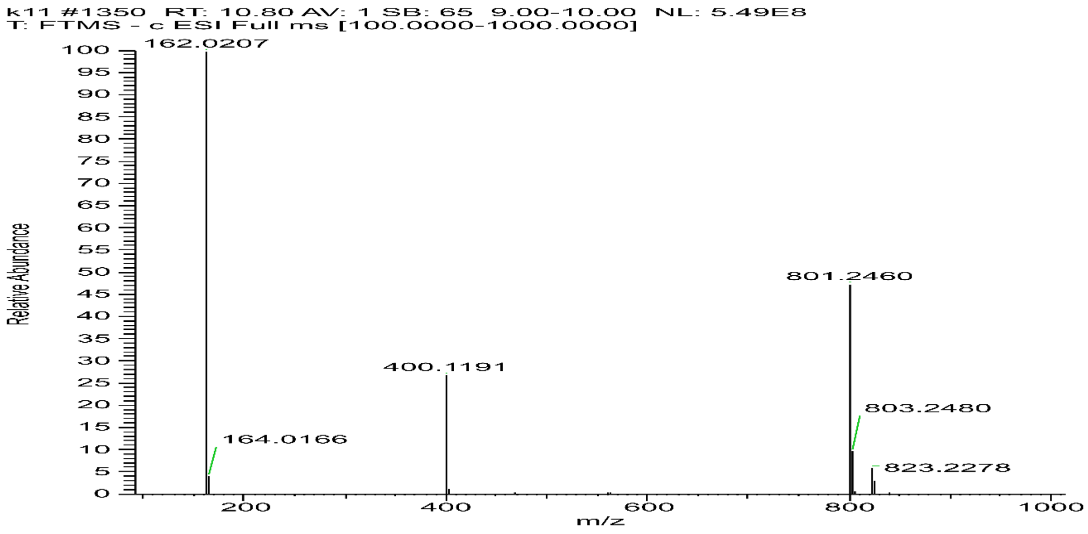

Figure S16. HPLC-UV-Vis spectrum of (*E*)(tR 16.354 min)/(*Z*)(tr 16.128 min) isomeric mixture of chalcone **1**.

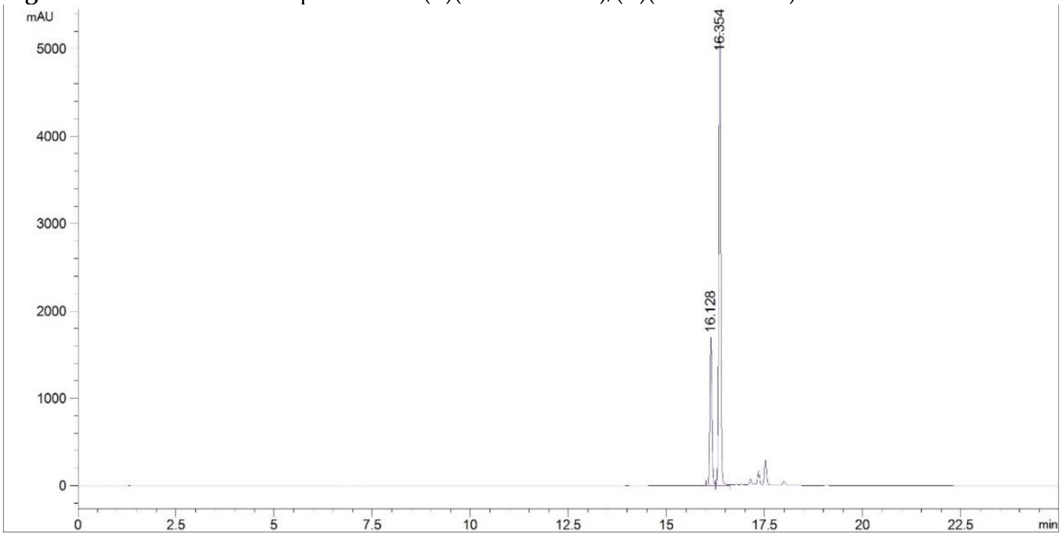

Figure S17. HPLC-UV-Vis spectrum of (*E*)(tR 15.607 min)/(*Z*)(tr 15.855 min) isomeric mixture of chalcone **2.**

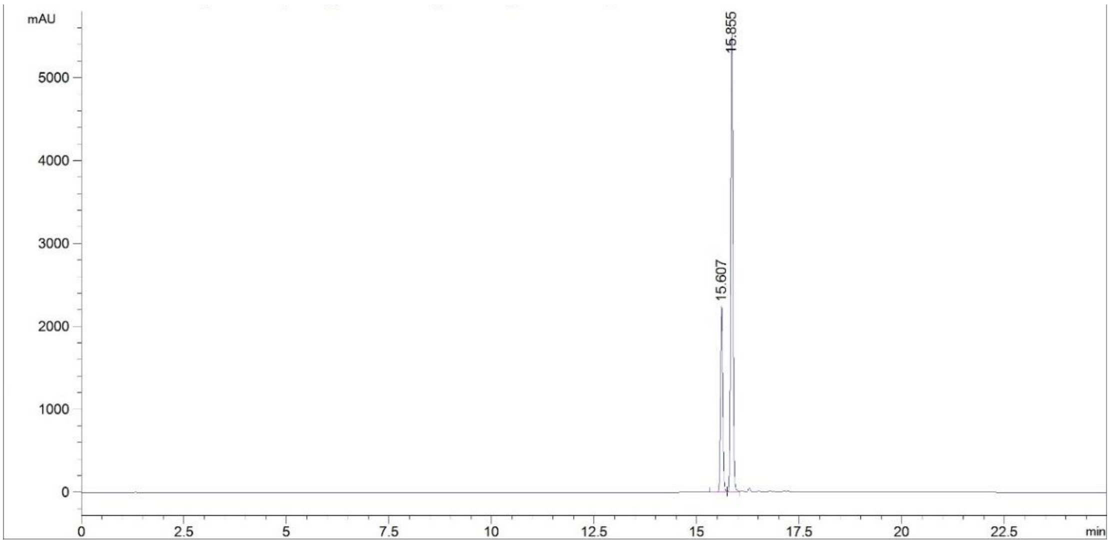

Figure S18. HPLC-UV-Vis chromatogram of pH 8.0/8.5 incubation (315-minute timepoint) of chalcone **1** and NAC.

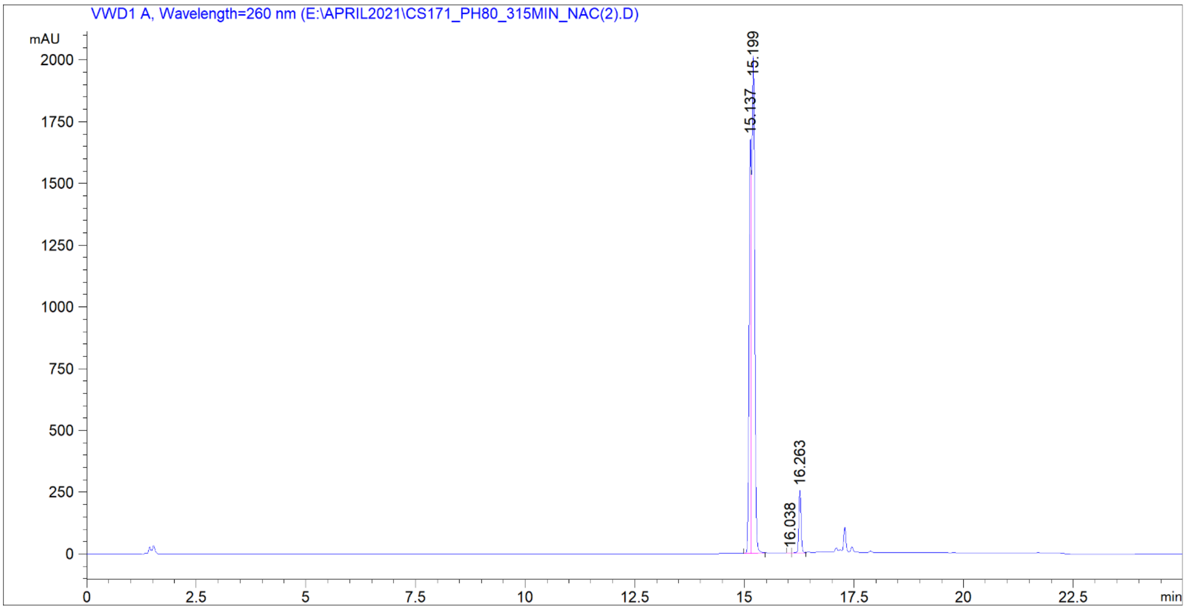

Figure S19. HPLC-UV-Vis chromatogram of pH 6.3/6.8 incubation (315-minute timepoint) of chalcone **1** and NAC.

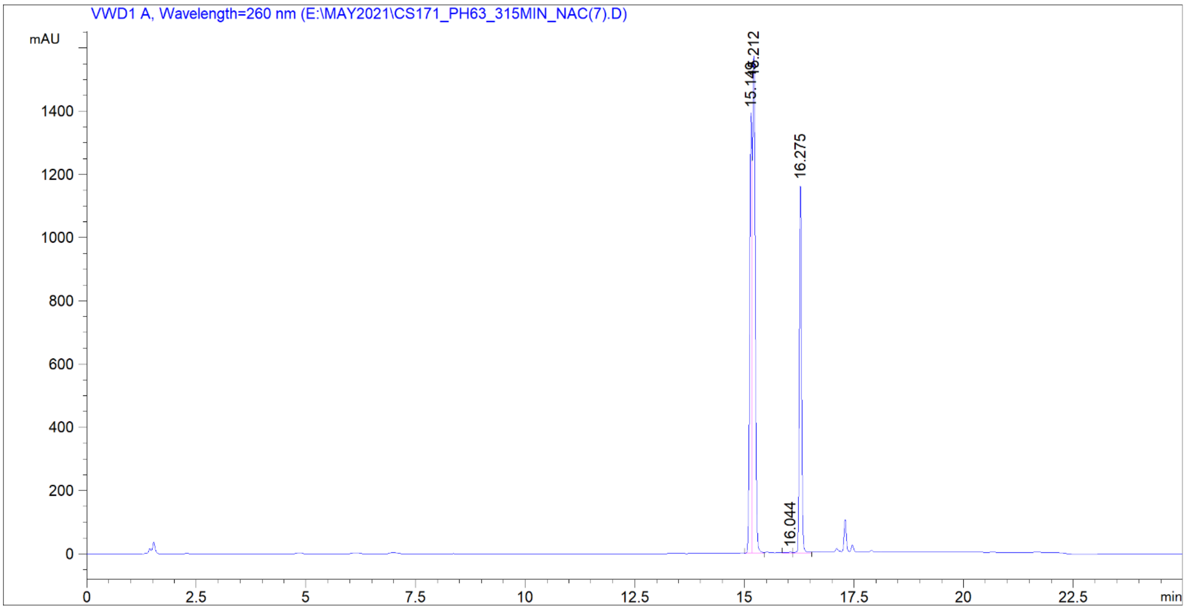

Figure S20. HPLC-UV-Vis chromatogram of pH 3.2/3.7 incubation (315-minute timepoint) of chalcone **1** and NAC.

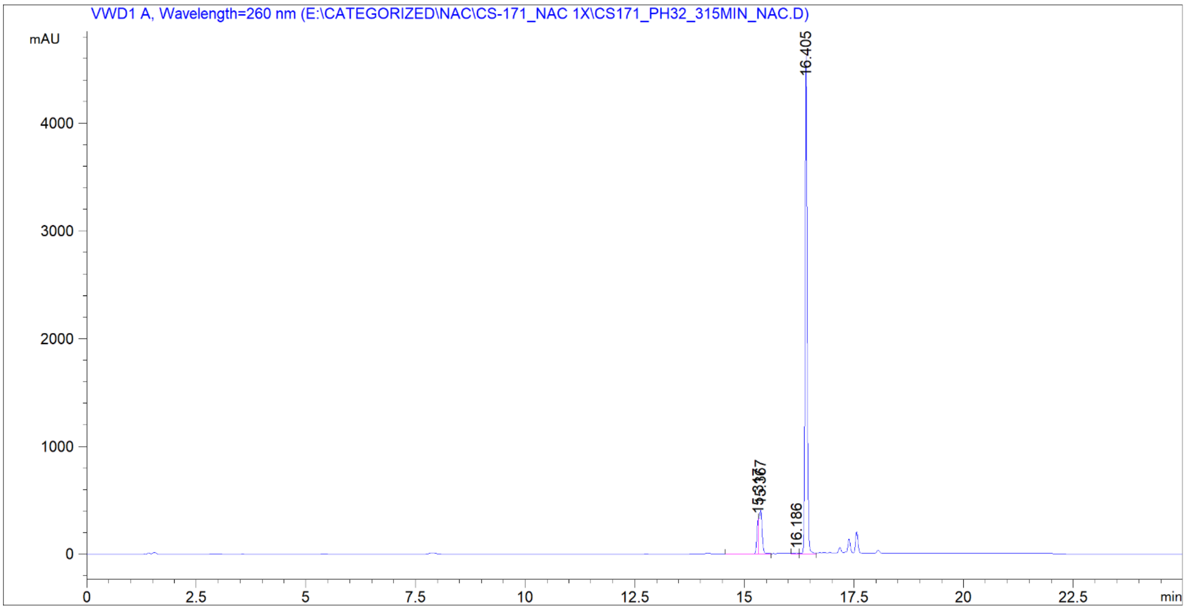

Figure S21. HPLC-UV-Vis chromatogram of pH 8.0/8.5 incubation (315-minute timepoint) of chalcone **2** and NAC.

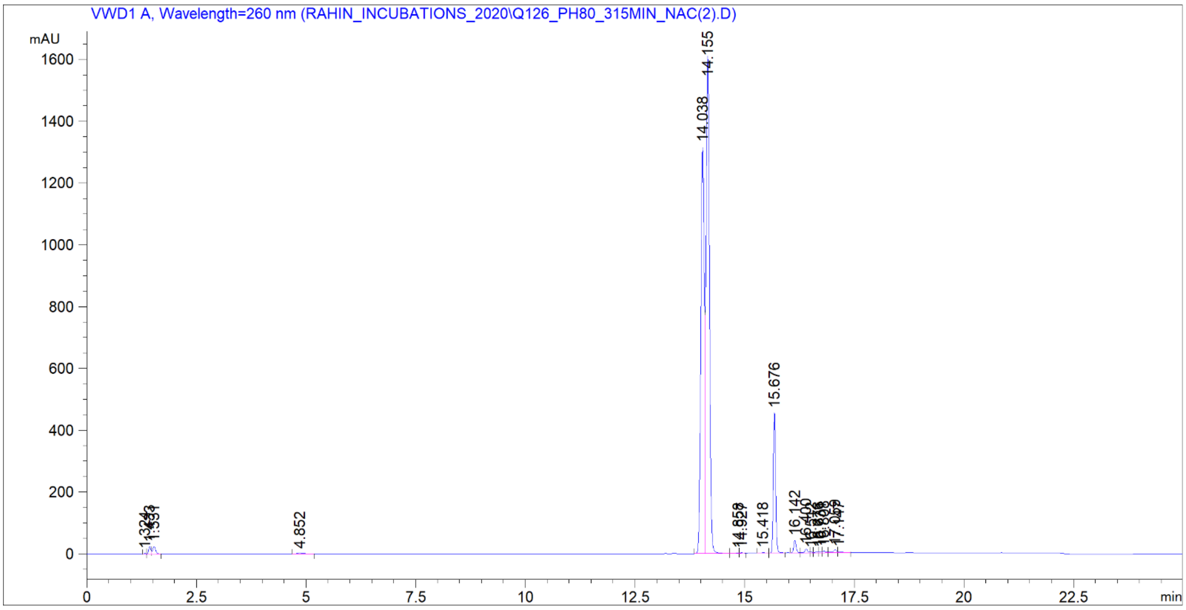

Figure S22. HPLC-UV-Vis chromatogram of pH 6.3/6.8 incubation (315-minute timepoint) of chalcone **2** and NAC.

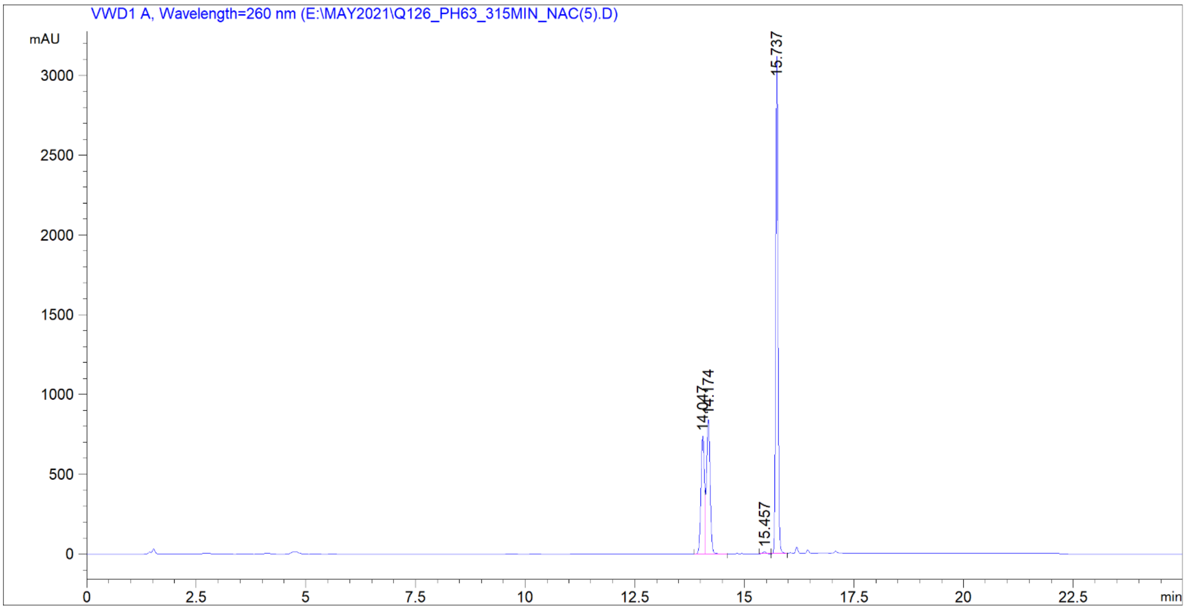

Figure S23. HPLC-UV-Vis chromatogram of pH 3.2/3.7 incubation (315-minute timepoint) of chalcone **2** and NAC..

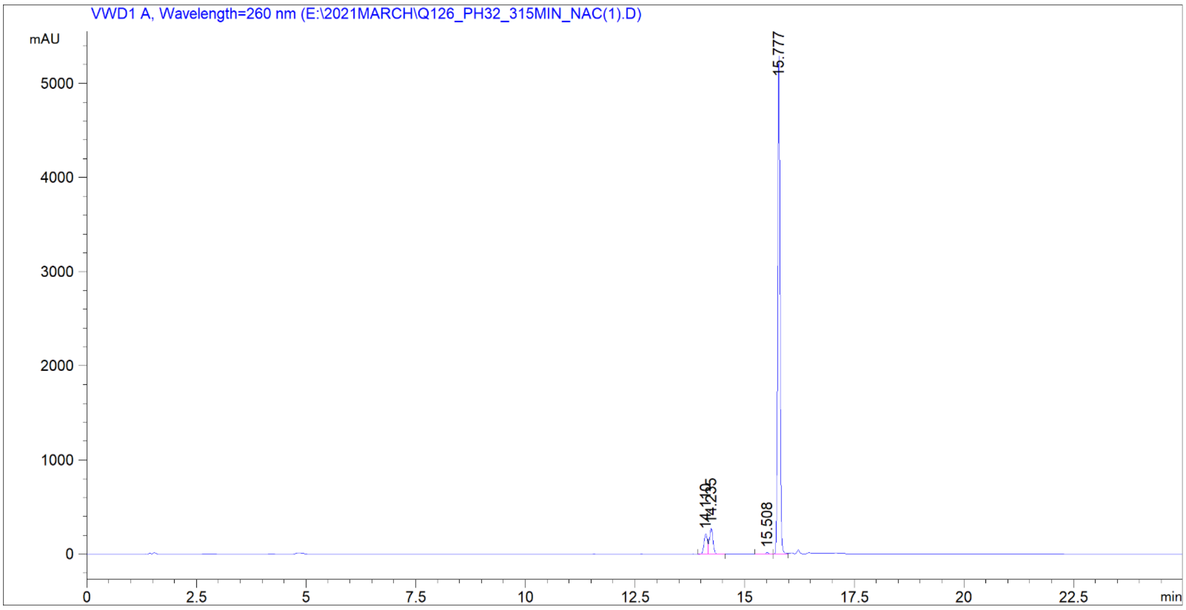

Supplement: Supplementary file 1 [file molecules-26-04332-s001.zip › molecules-1285113-SM-2nd proof_PP.pdf]
